# Investigating the Long-Term Effect of an Interdisciplinary Multimodal Rehabilitation Program on Levels of Bioactive Lipids and Telomerase Activity in Blood from Patients with Chronic Pain

**DOI:** 10.3390/jcm11051291

**Published:** 2022-02-26

**Authors:** Niclas Stensson, Björn Gerdle, Linn Rönne-Petersén, Liu L. Yang, Catharina Lavebratt, Torkel Falkenberg, Bijar Ghafouri

**Affiliations:** 1 Pain and Rehabilitation Centre, Department of Health, Medicine and Caring Sciences, Linköping University, 581 83 Linköping, Sweden; bjorn.gerdle@liu.se; 2 Department of Neurobiology, Care Sciences and Society, Division of Nursing, Karolinska Institutet, 171 77 Stockholm, Sweden; linn.ronne-petersen@ki.se (L.R.-P.); torkel.falkenberg@ki.se (T.F.); 3 Department of Molecular Medicine and Surgery, Karolinska Institutet, 171 77 Stockholm, Sweden; liu.yang@ki.se (L.L.Y.); catharina.lavebratt@ki.se (C.L.); 4 Center for Molecular Medicine, Karolinska University Hospital, 171 76 Stockholm, Sweden

**Keywords:** endocannabinoids, telomer length, telomerase activity, chronic pain, multimodal rehabilitation, physical activity

## Abstract

Mechanism-based diagnosis and therapies for chronic pain are lacking. However, bio-psycho-social interventions such as interdisciplinary multimodal rehabilitation programs (IPRPs) have shown to be relatively effective treatments. In this context we aim to investigate the effects of IPRP on the changes in levels of bioactive lipids and telomerase activity in plasma, and if these changes are associated with changes in pain intensity and psychological distress. This exploratory study involves 18 patients with complex chronic pain participating in an IPRP. Self-reports of pain, psychological distress, physical activity, and blood samples were collected before the IPRP and at a six-month follow-up. Levels of arachidonoylethanolamide (AEA) and 2-arachidonoylglycerol (2-AG), palmitoylethanolamide (PEA), oleoylethanolamide (OEA), stearoylethanolamide (SEA), and telomerase activity were measured. Pain intensity was decreased, and SEA levels were increased at the six-month follow up. A significant correlation existed between changes in SEA levels and pain intensity. AEA levels, were inversely correlated with physical activity. Furthermore, 2-AG and telomerase activity was significantly correlated at the six-month follow-up. This study confirms that IPRP is relatively effective for reduction in chronic pain. Changes in SEA were correlated with changes in pain intensity, which might indicate that SEA changes reflect the pain reduction effects of IPRP.

## 1. Introduction

Chronic pain is a frequent and distressing condition, often with a complex origin, that has a significant impact on individuals and society. The prevalence of chronic pain, according to data taken from population surveys, varies widely depending on case definition, and it is estimated (2006) that 19% of people in Europe have experienced pain of a moderate intensity for a duration of at least six months [1]. There are multiple risk factors for developing chronic pain, with female sex and higher age being the fixed risk factors [2], and lack of physical activity a variable risk factor [3]. Mental health aspects can also play a role, and depression and anxiety are related to both developing chronic pain and are regarded as comorbidities of chronic pain [4,5].

Interdisciplinary multimodal rehabilitation programs (IPRPs) are frequently used for the treatment of complex chronic pain. IPRP is a subgroup of interdisciplinary treatments according to the International Association for the Study of Pain (IASP). In Sweden, such biopsychosocial programs are typically based upon cognitive behavioural therapy (CBT, often including Acceptance and Commitment Therapy, ACT) and include physical exercise, pain education and return to work strategies. In comparison with usual care programs or “single-modal” approaches, IPRP has been reported with higher efficacy in decreasing pain and disability in chronic pain patients, according to systematic reviews [6,7]. These results have been confirmed in a large real-life study from the Swedish Quality Registry for Pain Rehabilitation (SQRP), which reported small to moderate effect sizes for the 22 mandatory outcomes [8].

Although IPRPs are the most efficient treatments for chronic pain patients today, there is a lack of mechanism-based approaches for diagnosis and treatment for chronic pain. According to the International Statistical Classification of Diseases and Related Health Problems-Tenth Revision (ICD-10), the diagnosis is based on the duration and anatomical location. Regarding treatment, patients are managed using a trial-and-error approach.

Expanded knowledge about ongoing molecular alterations in, e.g., pain/nociceptive, immune and stress systems in chronic pain patients is crucial for the development of mechanism-based diagnosis and treatment. One approach is to study how levels of endogenous molecules—involved in pain/nociception, stress, and immune systems—changes in response to common clinical treatments for chronic pain, e.g., IPRP. Additionally, the associations between clinical variables such as pain and depression with levels of endogenous molecules are important to investigate in chronic pain.

An endogenous system associated with multiple biochemical actions affecting, e.g., pain [9] inflammation [10], and psychological distress [11] is the endocannabinoid system (ECs). Moreover, this system has been reported to be acutely affected by physical activity [12], which is one of the components in IPRP.

The endocannabinoids (eCBs)—the lipid mediators of the ECs—are activators of the cannabinoid receptors (CB_1_ and CB_2_). Arachidonoylethanolamide (AEA) (also named anandamide) and 2-arachidonoylglycerol (2-AG) are both well-known eCBs. Meanwhile, other ethanolamide lipid mediators, namely the *N*-acylethanolamines (NAEs), do not activate CB receptors, but partially share metabolic enzymes with eCBs and have other molecular targets. Hence, palmitoylethanolamide (PEA) and oleoylethanolamide (OEA) can activate peroxisome proliferator activating receptor-α (PPAR-α) [13,14], and stearoylethanolamide (SEA) has been proposed to activate PPAR-γ [15].

The potential role ECs plays in various chronic pain conditions remains to be clarified, but emerging data suggest that this system may play an important role in the body’s descending pain pathways, and preclinical studies support that the eCBs are implicated in the control of pain initiation [16]. Furthermore, in healthy humans, acute aerobic exercise has been demonstrated to increase blood levels of eCBs and NAEs [17,18,19,20]. Concerning long-term effects of physical exercise in chronic pain patients, AEA was reported to be increased and SEA to be decreased after a 15-week resistant exercise program in women with fibromyalgia [21].

Another endogenous system associated with stress in humans is the telomer/telomerase complex [22]. Human studies of the telomere restoring enzyme telomerase, have shown increased telomerase activity (TA) after stress induction and that the increased activity was associated with increased cortisol levels. [23]. Moreover, telomere length (TL) has been shown to be positively associated with physical activity in humans [24]. Additionally, the TA has been proposed to be positively associated with physical activity, however this association seems to be specific to cell-type and/or genotype [25].

There are several studies that have reported the effectiveness of IPRP as a treatment approach in chronic pain [6]. Altered levels of eCBs and NAEs in patients with chronic pain have previously been reported [21,26,27]. To the best of our knowledge investigations about relationships between ECs/TA/TL and IPRP outcomes are lacking.

Hence, the aim of this explorative study is to investigate changes in these two endogenous systems associated with, e.g., pain, stress, and physical activity in patients after participating in an IPRP. Plasma levels of eCBs, and NAEs, as well as TL and TA, were collected from mononuclear cells before IPRP and at six months follow-up in patients with chronic pain, (who were referred to a multidisciplinary pain and rehabilitation centre at Linkoping’s university hospital). Within this aim, it is also investigated if levels and changes in these molecules are associated with changes in self-assessed pain intensity, psychological distress (depressive and anxiety symptoms) and physical activity levels, and correlations between levels of the eCBs/NAEs with TL and TA.

## 2. Materials and Methods

### 2.1. Study Design

This prospective study focusses on the longitudinal aspects of IPRP and investigates chronic pain patients before and after IPRP. IPRP was carried out for six weeks. Data of eCBs and NAEs in plasma and TL, TA from lymphocytes/monocytes, self-reports of pain, and depressive/anxiety symptoms and self-assessed physical activity levels were collected before (baseline), immediately after six weeks IPRP, and at a six-month follow-up. We chose to investigate the long-term effects of IPRP more specifically and the results presented in this work therefore focus on data collected before IPRP and at the six-month follow-up.

### 2.2. Patients, Clinical Examination, and IPRP

#### 2.2.1. Patients

Patients were referred to the Pain and Rehabilitation Centre, University Hospital, Linköping, Sweden by other healthcare services (mainly primary healthcare) for complex chronic non-malignant pain conditions. The Pain and Rehabilitation Centre in Linköping is associated with the Swedish Quality Registry for Pain Rehabilitation (SQRP). All clinical departments/centres within specialist care throughout Sweden deliver data to SQRP [28]. The SQRP is based on questionnaires and a detailed description of the SQRP has been reported previously [8,29]. During the first visit to the Pain and Rehabilitation Centre, all patients were given a clinical examination (see below) and completed the SQRP questionnaires. All patients provided informed consent to participate in the study and signed a consent form, which was in accordance with the Declaration of Helsinki. In addition to the questionnaires in the SQRP, blood samples were drawn from the patients.

The study was approved by the Regional Ethics Committee of Stockholm (Dnr: 2014/953-31/1).

#### 2.2.2. Clinical Examination

All patients were given a standard routine clinical examination performed by a physician. The examination consisted of a routine neurological examination, including registrations of systolic and diastolic blood pressures and auscultation of the heart and lungs. The use of medication, including analgesic drugs, was also reported: paracetamol, NSAID, opioids and antidepressants.

#### 2.2.3. IPRP

Medical assessments and decisions to offer IPRP were performed by senior physicians, primarily from specialists in rehabilitation medicine or similar specialties, or by specialists in training under the supervision of a senior colleague. Patients were also assessed by a psychologist, an occupational therapist, and a physiotherapist to be considered for the ability to carry out and complete IPRP. The following general inclusion criteria for IPRP were used: (i) disabling chronic pain (on sick-leave or experiencing major interference in daily life due to chronic pain); (ii) age between 18 and 67 years; (iii) no further medical investigations needed; (iv) written consent to participate and attend IPRP; (v) and agreement not to participate in other parallel treatments. General exclusion criteria included severe psychiatric morbidity, abuse of alcohol and/or drugs, diseases that did not allow physical exercise, or presence of red flags. Red flags are clinical indicators of possible serious underlying conditions requiring further medical intervention.

The IPRP was conducted in groups of six to nine patients for six weeks (at least 20 h per week of group-based activities) and was based on CBT (including ACT) in conjunction with physical exercise, pain education, return to work strategies, and pain education (including lectures in basic pain science and pain management both for patients as well as for relatives, friends, and colleagues). The program also included work-related advice and support, and individually tailored sessions with team members were available if necessary. Individual sessions might also be required for a few weeks following the IPRP. IPRP is mainly a group-based program but with opportunities for individual interventions, based on the clinical picture and the aims of the individual patient. The patient should be motivated and have the potential to make an active change. The individual’s expectations, preparation for changes, and how the pain has affected the possibility of activities were required to be clarified before starting treatment.

### 2.3. Self-Reports of Pain Intensity, Psychological Distress, Physical Activity, and BMI

#### 2.3.1. Pain Intensity

Numeric rating scale data (0 = no pain and 10 = worst possible pain) were collected for average pain intensity from the previous 7 days.

#### 2.3.2. Hospital Anxiety and Depression Scale

The Hospital Anxiety and Depression Scale (HADS), a short self-assessment questionnaire that measures symptoms of anxiety and depression, comprises seven items in each of the depression (HAD-D) and anxiety (HAD-A) scales [30]. HADS is frequently used in clinical practice, as well as in research, and has good psychometric characteristics [31] and is validated in its Swedish translation [32]. Scores range from 0 to 21, with the higher score indicating more severe depression and anxiety symptoms. A score of 7 or less indicates a non-case, a score of 8–10 indicates mild symptoms, and a score of 11 or more indicates a definite case.

#### 2.3.3. Physical Activity

The following indicator items, which were developed by the Swedish National Board of Health and Welfare, were used to register physical activity (PA).

PA1: During a typical week, how much time do you spend a week on physical exercise that makes you breathless, such as running, exercise gymnastics, or ball sports: 0 = 0–30 min; 1 = 30–60 min; 2 = 60–90 min; 3 = 90–120 min; or 4 = >120 min?

PA2: During a typical week, how much time do you spend a week on everyday exercise such as walking, cycling, or gardening: 0 = 0–30 min; 1 = 30–60 min; 2 = 60–90 min; 3 = 90–150 min; 4 = 150–300 min; or 5 = ≥ 300 min?

#### 2.3.4. Body Mass Index

The weight (kg) and height (m) were measured, and the BMI (kg/m^2^) was calculated.

### 2.4. Biological Measurements

#### 2.4.1. Blood Sampling

Blood samples were collected in EDTA-tubes and sodium citrate-tubes (BD Vacutainer^®^ CPT™ Mononuclear Cell Preparation, Franklin Lakes, NJ, USA). Samples were then centrifuged for the removal of red blood cells and the plasma fraction was transferred to new tubes, aliquoted in small portions, and stored at −86 °C. Lymphocytes and monocytes were prepared from the centrifuged citrate-tubes, according to manufacturer’s protocol. Half of the cells were snap-frozen for DNA extraction, and the rest were lysed by incubation with 120 µL CHAPS (Merck Millipore, Burlington, MA, USA) including 0.15 units/µL RiboLock (Thermo Fisher Scientific, Waltham, MA, USA) on ice for 30 min, followed by gentle vortexing. Samples were stored at −86 °C.

#### 2.4.2. Analysis of Bioactive Lipids

Levels of lipids were determined using liquid chromatography tandem mass spectrometry (LC-MS/MS) according to a previously published method [26]. Before the measurements, 300 μL of EDTA plasma were thawed and vortexed, and 30 μL of a mixture containing a deuterated internal standard (AEA-d4, OEA-d4, PEA-d4, and SEA-d3 (50 nM)) and 2AG-d5 (1000 nM) were added to each plasma sample. Lipids were then extracted using Octyl SPE columns (6 mL, 200 mg) (Biotage, Uppsala, Sweden) as described previously [28]. On the day of analysis, lipids were reconstituted in 30 μL of LC mobile phase A. The injection volume was 10 μL. An HPLC-MS/MS system containing a Thermo Scientific Accela AS auto sampler and Accela 1250 pump coupled to a Thermo Scientific TSQ Quantum Access max triple quadrupole mass spectrometer with a HESI II probe was used. LC was performed with a constant flow of 300 μL/min with mobile phase A, containing methanol-milliQ water-acetonitrile (4/4/2) (*v*/*v*/*v*) and mobile phase B containing methanol-acetonitrile (7/3) (*v*/*v*) with 0.1% (*v*/*v*) formic acid and 1 g/L ammonium acetate in A and B. Gradient elution was applied and started with 100% A during the first 1.5 min, followed by a linear increase towards 100% B, which was achieved after 9 min in total. Between the 11th and 12th min the gradient changed linearly to 100% A, which was maintained for 1 min. An Xbridge C8 analytical column (2.1 mm × 150 mm) with the particle size 2.5 µm obtained from Waters (Dublin, Ireland) was used. We used the following selected reaction monitoring (SRM) (*m*/*z*) transitions: 348.3/62.4; 326.3/62.4; 300.3/62.4; 328.3/62.4; and 379.3/287.3 for AEA, OEA, PEA, SEA, and 2-AG, respectively. For the corresponding internal standards, we used the following transitions: 352.3/62.4; 330.3/62.4; 304.3/62.4; 331.3/62.4; and 384.3/287.3 for AEA-d4, OEA-d4, PEA-d4, SEA-d3, and 2-AG-d5, respectively. The linearity of the analysis of each analyte was assessed with standard curves ranging from 1 to 25 nM for anandamide and 10 to 500 nM for OEA, PEA, and SEA, and 50 to 1250 nM for 2-AG in duplicate. The linearity of the standard curves was R^2^ ≥ 0.9 for all analytes. Isotopic dilution was used for quantification of the analytes, performed according to the area ratio of their corresponding deuterated internal standard signal area. Linear regression and X^2^ weighting were applied. Standards were purchased from Cayman Chemicals (Ann Arbor, MI, USA). Undetected levels were considered as 0 nM. Xcalibur^®^ (version 2.1, Thermo Scientific, Waltham, MA, USA) software was used for peak integration and quantification.

#### 2.4.3. TL Measurement

Genomic DNA was extracted according to protocol reported by O’Callaghan, N.J. and M. Fenech [33], using DNeasy^®^ Blood and Tissue Kit (Qiagene, Hilden, Germany) (with some modifications: the speed of centrifuge was changed to 6000× *g* and incubating was performed at 37 °C for 3 h instead of 56 °C for 10 min). DNA concentration was quantified with NanoDrop ND-1000 Spectrophotometer (Nano-Drop Technologies Inc., Wilmington, DE, USA). Relative TL was determined using real-time quantitative polymerase chain reaction (qPCR) according to [34], where the relative telomere to single copy gene (T/S) ratios was determined using a standard curve. In brief, each DNA sample (10 ng) was assessed for the telomere (*Tel*) and the single-copy gene (haemoglobin-b, *HGB)* in triplicate within the same 384-well plate, amplified by using Power SYBR Green in 10 µL total reaction volume. The reaction was performed on QuantStudio 7 Flex (Applied Biosystems; Life Technologies, Carlsbad, CA, USA) with the following conditions: 50 °C for 2 min, then 95 °C for 10 min, followed by 40 repeats of 95 °C for 15 s and 60 °C for 1 min, followed by a dissociation stage to monitor amplification specificity. The same standard curve of pooled DNA from these patient samples, ranging from 80 ng to 0.128 ng, was run on each plate for both genes and was used to determine the quantity of each gene for each sample. This allowed controlling for differences in the efficiencies between that of *Tel* and *HGB*. The gene quantities were then used to determine the T/S ratio for each sample. DNA samples with a Ct standard deviation of ≥0.35 between triplicates or a Ct value outside the standard curve were omitted from the analyses. The correlation coefficients of the standard curves were above 0.99 for each primer set and 384-plate. The inter-plate coefficient of variation (CV) of T/S ratio was 9.1% calculated from a patient sample run in seven 384-well plates. The TL analysis detection success rate was 100%. The primer sequences were (written 5′à3′): Tel1: CGGTTTGTTTGGGTTTGGGTTTGGGTTTGGGTTTGGGTT; Tel2: GGCTTGCCTTACCCTTACCCTTACCCTTACCCTTACCCT; *HGB* Fw: GCTTCTGACACAACTGTGTTCACTAGC; *HGB* Rv: CACCAACTTCATCCACGTTCACC. Samples from all three timepoints per person were assayed in the same 384-well plate.

#### 2.4.4. TA Measurement

The TA was measured using real-time telomeric repeat amplification protocol (RT-TRAP), using a modified protocol described by Hou et al. [35]. Briefly, the cell lysate was thawed and centrifuged at 4 °C at 12,000× *g* for 20 min. The supernatant was transferred to a new tube and total protein concentration was measured using a DC Protein Assay (Bio-Rad, Hercules, CA, USA). A volume of each sample containing 3.8 µg of total protein was analysed by PCR using the primers TS (5′-AATCCGTCGAGCAGAGTT-3′) and ACX (5′-GCGCGG(CTTACC)3CTAACC-3′). TSR8, an oligonucleotide with a sequence identical to the TS primer extended with 8 telomeric repeats being AG(GGTTAG), was used to generate a standard curve. The amplification reaction was terminated and 8 µL of the telomeric repeat products were used for the RT-TRAP assay amplified by 8 µL Power SYBR Green in 384-well plates. The analysis was performed on QuantStudio 7 Flex (Applied Biosystems; Life Technologies; Thermo Fisher Scientific Inc.) with the following conditions: 95 °C for 10 min, followed by 36 repeats of 95 °C for 20 s, 52 °C for 30 s and 72 °C for 60 s. All samples and standards were analysed in triplicates.

### 2.5. Statistical Analysis

A comparison was made between data from baseline (before IPRP) and six months after IPRP, as the focus of this study was to investigate the long-term effect of IPRP. However, data from baseline and direct after IPRP were analysed and included as a Supplementary File. Paired sampled *t*-tests were used for comparison of data between timepoints as the data was shown to be normally distributed using Shapiro–Wilk test. Group mean with standard deviations are presented. Bivariate correlations were analysed using Pearson’s. *p* ≤ 0.05 was used as level of significance in all statistical analyses. Data analyses were performed using IBM SPSS (version 26.0; IBM Corporation, Route 100 Somers, New York, NY, USA) and GraphPad Prism (version 9.0.0; GraphPad Software Inc., San Diego, CA, USA).

## 3. Results

Eighteen patients (13 women and 5 men) with 12 different diagnoses (Table 1), a mean (SD) age of 46.4 (10.7) years and a mean of pain duration of 6.5 (7.2) years were included in this study. This study has an exploratory nature; therefore, no sample size was calculated. Based on our earlier study on the same cohort [5] investigating inflammatory proteins before and 12 months after IPRP, we concluded that the numbers of subjects should be enough and will not have problems with power. About 55% of the participants were taking prescription drugs. Details about the prescription drug at onset and at final evaluation are presented in Supplementary File S1. The patient’s main diagnosis was determined using the Swedish version of ICD-10 (ICD10-SE); the main diagnoses are listed in Table 1. The results presented are analysed data from the time before IPRP and from the six-month follow-up. However, data from the time immediately after IPRP are enclosed as Supplementary File S2.

### 3.1. Pain Intensity, Psychological Distress, Physical Activity, and BMI

Pain intensity scorings were significantly decreased from baseline to follow up six months after IPRP. For HAD-A: at baseline, nine patients were rated as non-cases (0–7), two patients as borderline cases (8–10) and seven patients as definitive cases (11–21). At follow-up, the number of non-cases had increased to twelve, borderline cases to three and definitive cases decreased to three. For HAD-D: at baseline, seven patients rated themselves as non-cases (0–7), eight patients as borderline cases (8–10) and three patients as definitive cases (11–21). At follow-up, the number of non-cases had increased to ten, borderline cases had decreased to 4 and definitive cases had increased to four. No statistically significant changes in HAD scores or physical activity levels (PA1 and PA2) were reached on group level six months after IPRP, however a tendence of lower HAD values and higher PA scores could be observed at the six-month follow-up (Table 2). No significant changes in BMI existed between the two occasions (BMI (mean (SD)) baseline IPRP: 27.0 (3.8) at the six-month follow-up: 26.5 (3.8)).

### 3.2. Biological Measurements

#### Levels of Bioactive Lipid Mediators

Plasma SEA levels were significantly increased at follow up compared to baseline (*p* = 0.04). Concentrations of the lipids before and after the IPRP are presented in Table 3.

### 3.3. TL and TA

No significant changes between baseline and follow-up were found for either TL (T/S ratio) (baseline: 1.24 (0.65); follow-up: 1.36 (0.56)) or TA (TPG units/µL) (baseline: 1.49 (1.1); follow-up: 1.47 (0.73)).

### 3.4. Correlation Analyses

Correlations between, on the one hand self-reported pain intensity, physical activity, psychological distress, and on the other hand, the lipid mediators (AEA, 2-AG, OEA, PEA, SEA), TL and TA were analysed at baseline and at the six-month follow-up. Correlations analyses were also performed on the calculated change (baseline minus follow up = ∆) of these variables (Table 4). Age and BMI did not associate with any of the analytes and, hence, were not adjusted for.

**Figure 1:**
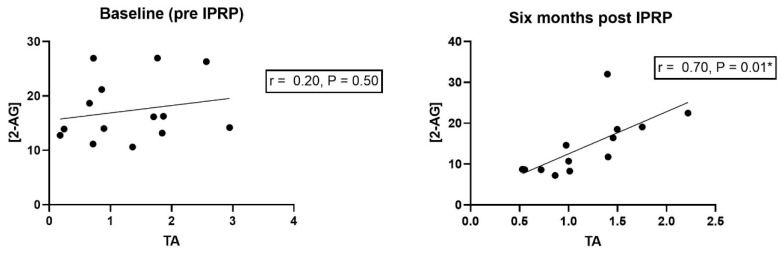
Scatter plots with best fitted regression lines of 2-arachidonoylglycerol (2-AG) and telomerase activity (TA) pre (**left** panel), and six months post interdisciplinary multimodal rehabilitation program IPRP (**right** panel). r = Pearson’s correlation coefficient and * indicate statistical significance.

At baseline, significant correlations existed between: PA1 and AEA (r: −0.49, *p*: 0.05) (Table 4) and OEA (r: −0.56, *p*: 0.02). At the follow-up, significant correlations existed between: PA2 and AEA (r: −0.56, *p*: 0.04) (Table 4), and TA and levels of 2-AG (r: 0.70, *p* = 0.001). In Figure 1 scatter plots of TA and 2-AG are presented before IPRP and at six months follow-up.

Significant ∆-correlations existed between ∆SEA and ∆Pain intensity (r: −0.47, *p* = 0.05), ∆SEA and ∆TL (r: −0.52, *p* = 0.03), and interestingly between ∆AEA and ∆PA (1 and 2) (r: −0.50, *p* = 0.04 and r: −0.58, *p* = 0.01, respectively) (Table 4). (Tables of all correlations before the IPRP, at the six-month follow-up, and ∆-correlations exist in Supplementary File S3).

## 4. Discussion

The key findings in this study are as follows:

- SEA levels were significantly increased at the six-month follow-up, and the changes were inversely correlated with changes in pain intensity;
- AEA and physical activity levels were inversely correlated at baseline and at the follow-up, and moreover, changes in AEA were inversely correlated with changes in physical activity;
- A strong positive correlation existed between levels of 2-AG and TA at the six-month follow-up.

Six months after attending an IPRP, pain intensity was reduced (*p* = 0.05) in patients with complex chronic pain: with a moderate Cohen’s d effect size (0.46). This was in line with a large study from the Swedish Quality Registry for Pain Rehabilitation, which reported small to moderate effect sizes for 22 mandatory outcomes, including pain intensity [8], confirming that IPRP have a long-term pain reducing effect for chronic pain patients. Anxiety and depression scorings were decreased at the six-month follow-up; however, statistical significance level was not reached, yet the mean anxiety scoring changed (Cohen’s d = 0.43) from 7.7 (indicating mild symptoms) to 5.3 (no symptoms) (*p* = 0.06) (Table 2) and can be considered as borderline significant.

Patients reported higher levels of physical activity at the six-month follow-up compared with pre the IPRP program. PA1 (physical activity that causes shortness of breath) scorings were especially higher at follow-up compared with pre IPRP. However, this increase was not statistically significant (*p* = 0.11), yet Cohen’s d was 0.59, which may indicate that a substantial increase in aerobic exercise was initiated by the IPRP and that this increase was maintained six months after the program completed.

Concerning the eCB and NAE levels, no significant differences existed except for SEA, which was increased significantly at the six-month follow-up (*p* = 0.04), and moreover, concentration changes in SEA correlated negatively with changes in pain intensity (r = −0.47, *p* = 0.05).

Pre-clinical studies have reported SEA with anti-inflammatory, cytokine regulating [15,36] characteristics; however, a more recent report highlighted PEA and OEA—not SEA—with these attributes [37]. If compared with the previous findings from human studies, SEA was significantly decreased in women with fibromyalgia (*n* = 37) after a 15-week strength training intervention and pain intensity was significantly decreased [21]; the result from this study is the reverse. Possible explanations for these contrasting results include different diagnosis, different interventions and that the included subjects in this study are real-life patients who were referred to the rehabilitation clinic because of their pain. This study includes patients with high heterogeneity (different diagnoses and both sexes) who have been included in IPRP based on the clinical picture, and the aims of the individual patient, how motivated they are, and if they have potential for an active change, while the patients recruited for this research study have been selected based on other criteria. Therefore, the result from this study can be compared to only those studies that are very limited, who have investigated “real-life patients”. The patients in this study have conducted specific individual exercises with a physiotherapist (with both relaxation therapy and strengthening specific regions of the body to unburden), individual- and group therapy led by a clinical psychologist (cognitive behavioural therapy, education, coping and mindfulness), as well as environmental work changes from an occupational therapist, working with education and ergonomic tools to alleviate and prevent painful situations, for six weeks (at least 20 h per week of group-based activities). In the study by Stensson et al. [21] the intervention was based only on strength training and the other component of the IPRP was not included. Perhaps one can hypothesise that there are different neurobiological mechanisms that might be activated, independent of diagnosis, and that in turn could be an explanation for the lack of positive effect of IPRP for certain chronic pain states. Larger studies with homogenous chronic pain states are warranted to be able to generalize the result in this study.

Acute aerobic exercise has consistently been reported to increase AEA in blood in healthy humans [16,17,18,38]. Regarding the inverse association between AEA levels and physical activity in the present study, the trend was clear. Inverse correlations existed between AEA levels and PA1 (r = −0.49) before the IPRP program and with PA2 (r = −0.56) at the six-month follow-up, which indicates that a higher physical activity level lowers the basal plasma tone of AEA. Moreover, ∆AEA correlates with both ∆PA1 and ∆PA2 (r = −0.50 and r = −0.58) (Table 4), which implies that larger increases in physical exercise have a higher impact on decreasing plasma concentrations of AEA. This result is in agreement with Brellenthin et al. who reported a significant inverse association between self-reported physical activity levels and AEA concentrations [38], partly in-line with Belitardo de Oliveira et al.’s report of decreased plasma AEA levels after 12 weeks of aerobic exercise in healthy people [19]. However, no association between physical activity and AEA was reported in that study (and in our present study a tendency of increased AEA exist) (Table 3). It was also partly in line with Stensson et al.’s report of increased AEA after a 15-week resistant exercise program in women with fibromyalgia [21]; however, no association between AEA and physical activity was reported in that study either. Possible explanations for an inverse relationship between blood levels of AEA and habitual physical activity levels could be dependent on fatty acid amide hydrolase—an enzyme crucial for degrading AEA—whose activity in lymphocytes compared to sedentary human subjects have been reported to be significantly higher [39]. However, more studies are needed to reveal the exact relationship between physical exercise and AEA.

Concerning the strong correlation between 2-AG and TA at the six-month follow-up, to the best of our knowledge this is the first study reporting about this relationship, hence the result needs to be interpreted with caution. However, if this dependence is valid, one possible link could be via corticosteroids. Cortisol has been demonstrated to decrease TA in human T lymphocytes [40]. However, in response to experimental stress in humans, Epel et al. reported that TA was positively associated with cortisol, but with a delayed onset compared to cortisol [23]. Corticosterone treatment has been reported to increase 2-AG levels in rat brain [41,42]. One can hypothesize that since this study measures levels of 2-AG and TA in circulation, IPRP might contribute to homeostatic effects in the 2-AG–TA–glucocorticoid relationship, however this result needs to be further investigated in future studies.

The main limitations of this study are the small cohort size (18 patients), the single-armed design, and the heterogeneous patient group. The present biochemistry findings may be common for chronic pain in general but in future larger studies it is necessary to investigate and confirm the results in different diagnoses and mechanisms (nociceptive, neuropathic and nociplastic). Larger studies that include a control group are needed to confirm the validity of our findings. The weakness of self-reported values should also be added here, where multiple factors can play a role in both overestimating and underestimating the level of one’s own physical activity, objective measures, e.g., VO_2_MAX would have increased the validity of the results. Diet has been reported to influence eCBs and NAE’s, as well as TL and TA, and was not controlled for in this study. AEA levels vary during the menstrual cycle and peak in the ovulation phase [43]. This was not controlled for in this study, neither were pre vs. post menopause effects on lipid levels controlled for.

## 5. Conclusions

This study confirms that IPRP relieves pain and may reduce psychological distress in patients with chronic complex pain. When it comes to biochemistry, levels of SEA were increased six months after the program, and baseline to follow-up changes in SEA levels were associated with changes in pain intensity, which may indicate that SEA level changes reflect aspects of the positive pain reduction effects of IPRP. An inverse relationship between self-reports of physical activity levels and AEA levels existed in these patients, and a possible link between the endocannabinoid system and telomerase/telomer complex was detected; however, more studies are needed to confirm the validity of these results.

## Figures and Tables

**Table 1:** Main diagnosis code (ICD-10-SE) of the 18 patients.

Diagnose Code	Denotation	Number of Patients
M35.7	Hypermobility syndrome	1
M53.1	Cervicobrachial syndrome	1
M54.4	Lumbago with sciatica	3
M54.5	Low back pain	2
M54.6	Pain in thoracic spine	1
M54.8	Other dorsalgia	1
M79.1	Myalgia	1
M79.7	Fibromyalgia	3
R52.2A	Chronic pain, nociceptive	2
R52.2B	Chronic pain, neuropathic	1
R52.2C	Other chronic pain	1
T91.8A	late discomfort due to Whiplash	1

**Table 2:** Mean (SD) for pain intensity, hospital anxiety and depression scale (HAD-A and HAD-D, respectively), physical activity (PA 1 and PA 2) at baseline and six months after the interdisciplinary multimodal rehabilitation program. * Indicate statistically significance.

Scale	Baseline	Six-Month Follow Up	*p*-Value
Pain intensity	6.83 (1.29)	6.11 (1.74)	0.05 *
HAD-A	7.67 (5.55)	5.33 (5.20)	0.06
HAD-D	7.00 (3.74)	5.61 (5.14)	0.14
PA 1	1.79 (1.63)	2.86 (1.96)	0.11
PA 2	2.64 (1.28)	2.93 (1.44)	0.37

Note: Comparisons between baseline and follow-up were performed with paired sample *t*-tests.

**Table 3:** Mean (SD) concentrations of arachidonoylethanolamide (AEA), 2-arachidonoylglycerol (2-AG), palmitoylethanolamide (PEA), oleoylethanolamide (OEA), stearoylethanolamide (SEA) before the interdisciplinary multimodal rehabilitation program and at a six-month follow-up. * Indicate statistically significance.

Lipid (nM)	Baseline	Six-Month Follow Up	*p*-Value
AEA	0.77 (0.48)	1.08 (0.61)	0.15
2-AG	15.80 (5.96)	14.60 (6.98)	0.58
OEA	5.29 (1.66)	5.74 (1.50)	0.36
PEA	4.67 (1.30)	4.83 (1.13)	0.66
SEA	3.90 (2.59)	5.10 (2.92)	0.04 *

Note: Comparisons between baseline and follow-up were performed with paired sample *t*-tests.

**Table 4:** Correlations between AEA levels and self-reported physical activity before IMMRP (*Pre*) and at a six-month follow-up (*Post*), and between the differences (*Pre-Post*) (∆AEA and ∆PA1 ∆PA2) * denote statistical significance.

	PA1 *Pre*	PA1 *Post*	PA2 *Pre*	PA2 *Post*	∆PA1	∆PA2
AEA *Pre*	−0.49 *		−0.10			
AEA *Post*		−0.45		−0.56 *		
∆AEA					−0.50 *	−0.58 *

Note: Pearson correlation coefficients are reported.

## Data Availability

The datasets generated and/or analysed in this study are not publicly available as the Ethical Review Board has not approved the public availability of these data.

## Supplementary Materials

The following are available online at https://www.mdpi.com/article/10.3390/jcm11051291/s1, Files S1–S3.
