# Supplementary material for: Investigating the Long-Term Effect of an Interdisciplinary Multimodal Rehabilitation Program on Levels of Bioactive Lipids and Telomerase Activity in Blood from Patients with Chronic Pain
Source: J Clin Med. 2022 Feb 26;11(5):1291. doi: 10.3390/jcm11051291 (PMC8911430; doi:10.3390/jcm11051291)
Supplement: Supplementary file 1 [file jcm-11-01291-s001.zip › Supplementary file S1.pdf]

Supplementary File S1. List of prescription drug for patients before IPRP and after 6 months attending IPRP.

**Before IPRP**	Pat 1	Pat 2	Pat 3	Pat 4	Pat 5	Pat 6	Pat 7	Pat 8	Pat 9	Pat 10
**Drug**	**Paracetamol**	**Paracetamol**	**Paracetamol**	**Paracetamol**	**Buprenorphine**	**Paracetamol**	**Paracetamol**	**Paracetamol**	**Paracetamol+codeine**	**Paracetamol**
Doses	665mg x6	2x 665 mg x3	500mg x4	665mg x 6	10mg/v	665mg x 6	665mg x 6	1g x 5	500mgx1-2	665mgx2-3
Days (last week)	7	7	7	7	7	7	7	7	4	7
**Drug**	**Naproxen**		**Chlorzoxazone**	**Duloxetin**	**Paracetamol**	**Duloxetine**	**Naproxen**		**Amitriptyline**	**Naproxen**
Doses	250mg x2		250mg x 4	90mg x 1	665mg x 3	60mg x 1	500mg x 2		10mgx6	500mgx2
Days (last week)	1		7	7	7	7	7		7	7
**Drug**	**Omeprazole**			**Oxycodone**		**Etoricoxib**	**Chlorzoxazone**			**Codeine**
Doses	20mg x 1			10mg x 2		60mg x 1	50mg x 3			25mgx1-2
Days (last week)	1			7		7	7			7
**Drug**				**Oxycodone**						
Doses				5mg x 2						
Days (last week)				7						

**6 months after IPRP**	Pat 1	Pat 2	Pat 3	Pat 4	Pat 5	Pat 6	Pat 7	Pat 8	Pat 9	Pat 10
**Drug**	**Paracetamol**	**Paracetamol**	**Paracetamol+codeine**	**Paracetamol**	**Buprenorphine**	**Chlorzoxazone**	**Diclofenac**	**Pregabalin**	**Amitriptyline**	**Metoprolol**
Doses	665mg x 6	665 mg 2 x 3	500/30mg x 2	665mg x 6	10mg	3x1	10mg 2x2	50mgx2	1x1	50mg x 1

Days (last week)	7	7	3	7	7	7	7	4	6	7
**Drug**	**Naproxen**			**Duloxetine**	**Paracetamol**	**Paracetamol**	**Paracetamol**		**Paracetamol**	
Doses	250mg			90mg x 1	665mgx2x4	665mg x 6	1mg 2x2		2x3	
Days (last week)	2			7	7	7	7		2	
**Drug**					**0**	**Naproxen**	**Paracetamol+codeine**			
Doses						500mg x 6	500/30mg x1			
Days (last week)						7	7			
**Drug**						**Naproxen**				
Doses						250mg x 2				
Days (last week)						7				
