# Supplementary material for: Investigating the Long-Term Effect of an Interdisciplinary Multimodal Rehabilitation Program on Levels of Bioactive Lipids and Telomerase Activity in Blood from Patients with Chronic Pain
Source: J Clin Med. 2022 Feb 26;11(5):1291. doi: 10.3390/jcm11051291 (PMC8911430; doi:10.3390/jcm11051291)
Supplement: Supplementary file 1 [file jcm-11-01291-s001.zip › Supplementary file S2.pdf]

Supplementary file S2

Data from the investigated chronic pain subjects is presented from three different occasions where timepoint 1 = before the interdisciplinary multimodal rehabilitation program (IPRP), timepoint 2 = immediately after IPRP, and timepoint 3 = six-month follow-up. Levels of the bioactive lipids arachidonoylethanolamide (AEA) and 2-arachidonoylglycerol (2-AG), palmitoylethanolamide (PEA), oleoylethanolamide (OEA), stearoylethanolamide (SEA), are presented in **figure S1.** Telomere length (TL) telomerase activity (TA) are shown in **figure S2.** In **figure S3** self-reports of physical activity (PA) 1 and 2, psychological distress (HAD-A, HAD-D), and pain intensity (NRS) are presented.

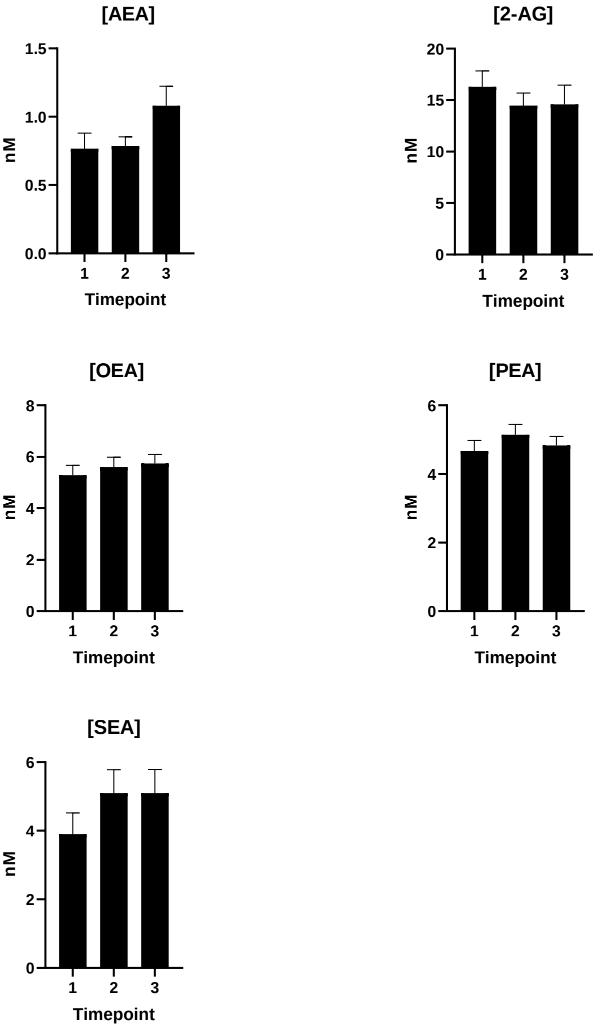

Figure S1. Mean levels with error bars in SEM of arachidonoylethanolamide (AEA) and 2-arachidonoylglycerol (2-AG), palmitoylethanolamide (PEA), oleoylethanolamide (OEA), stearoylethanolamide (SEA) are presented in nano molar (nM).Timepoint 1=before IPRP, 2 =immediately after IPRP, 3= six months follow-up.

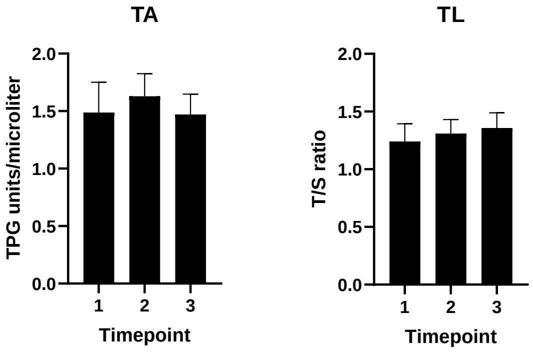

Figure S2. Mean levels of telomerase activity (TA) and Telomere length (TL). Timepoint 1=before IPRP, 2 =immediately after IPRP, 3= six months follow-up.

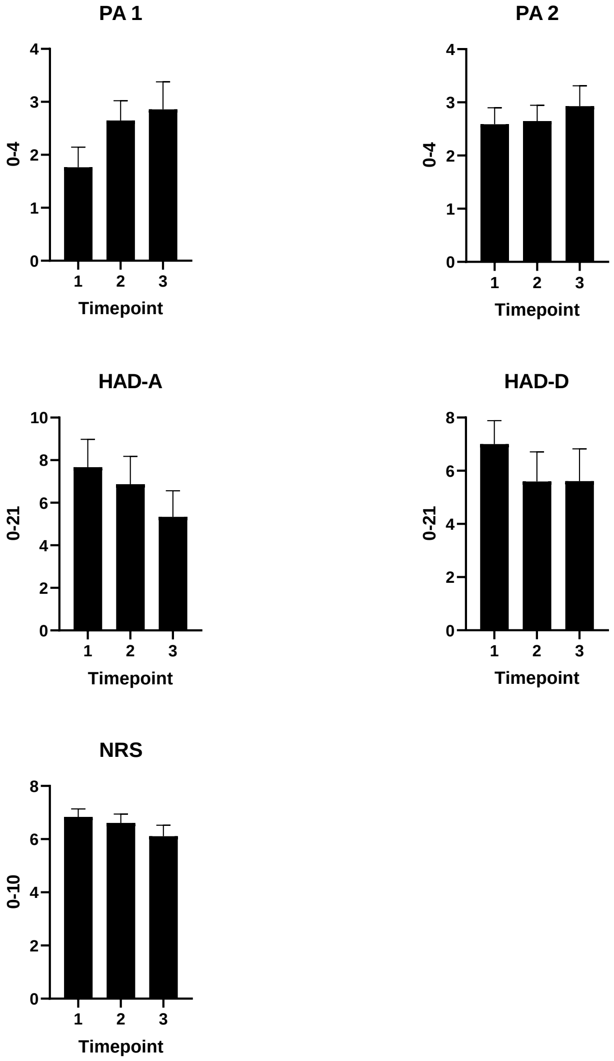

Figure S3. Mean levels of self-reports of physical activity (PA 1= exercise that makes you breathless), (PA 2= easier exercise such as walking and gardening. psychological distress (HAD-A =hospital anxiety scale, HAD-D =hospital depression scale), and pain intensity (NRS). Timepoint 1=before IPRP, 2 =immediately after IPRP, 3= six months follow-up.
