# Supplementary material for: Investigating the Long-Term Effect of an Interdisciplinary Multimodal Rehabilitation Program on Levels of Bioactive Lipids and Telomerase Activity in Blood from Patients with Chronic Pain
Source: J Clin Med. 2022 Feb 26;11(5):1291. doi: 10.3390/jcm11051291 (PMC8911430; doi:10.3390/jcm11051291)
Supplement: Supplementary file 1 [file jcm-11-01291-s001.zip › Supplementary file S3.pdf]

Supplementary Table S1 - Bivariate correlations before the interdisciplinary multimodal rehabilitation program

**Measure**		Age	BMI	NRS	HADA	HADD	PA1	PA2	AEA	2-AG	OEA	PEA	SEA	TL	TA
Age	Pearson Correlation	1	0.25	0.21	-0.01	0.06	-0.46	0.26	0.04	-0.23	0.37	0.41	0.41	-0.31	-0.35
	Sig. (2-tailed)		0.30	0.41	0.96	0.81	0.06	0.32	0.87	0.39	0.13	0.09	0.09	0.20	0.18
BMI	Pearson Correlation		1	0.03	0.09	-0.07	-0.06	0.19	-0.20	-0.05	0.27	0.24	-0.11	0.22	-0.39
	Sig. (2-tailed)			0.89	0.72	0.77	0.82	0.47	0.43	0.84	0.27	0.33	0.66	0.39	0.13
NRS	Pearson Correlation			1	-0.02	-0.11	0.16	-0.14	-0.39	0.19	-0.19	-0.28	0.3	0.07	-0.34
	Sig. (2-tailed)				0.95	0.67	0.55	0.60	0.11	0.48	0.46	0.27	0.16	0.78	0.20
HADA	Pearson Correlation				1	0.60**	0.07	-0.24	0.03	-0.13	0.00	-0.02	-0.01	-0.30	-0.40
	Sig. (2-tailed)					0.01	0.78	0.35	0.90	0.63	0.99	0.92	0.95	0.23	0.12
HADD	Pearson Correlation					1	-0.31	-0.08	0.06	-0.21	0.27	0.23	0.23	-0.37	-0.37
	Sig. (2-tailed)						0.23	0.75	0.82	0.44	0.28	0.35	0.36	0.13	0.16
PA1	Pearson Correlation						1	0.14	-0.49*	0.35	-0.56*	-0.35	-0.17	0.39	0.14
	Sig. (2-tailed)							0.60	0.04	0.20	0.02	0.16	0.52	0.12	0.61
PA2	Pearson Correlation							1	-0.10	-0.28	0.14	0.21	-0.18	-0.3	0.16
	Sig. (2-tailed)								0.70	0.31	0.60	0.42	0.49	0.31	0.5
AEA	Pearson Correlation								1	-0.12	0.23	0.57*	0.21	-0.12	0.30
	Sig. (2-tailed)									0.67	0.35	0.01	0.39	0.62	0.25
2-AG	Pearson Correlation									1	0.09	0.07	-0.02	0.65**	0.19
	Sig. (2-tailed)										0.74	0.79	0.93	0.01	0.50
OEA	Pearson Correlation										1	0.58*	-0.06	-0.11	-0.34
	Sig. (2-tailed)											0.01	0.81	0.67	0.20
PEA	Pearson Correlation											1	0.30	0.12	0.13
	Sig. (2-tailed)												0.23	0.64	0.64
SEA	Pearson Correlation												1	-0.18	-0.24
	Sig. (2-tailed)													0.47	0.36
TL	Pearson Correlation													1	0.19
	Sig. (2-tailed)														0.49
TA	Pearson Correlation														1

Supplementary Table S2 - Bivariate correlations at the six-month follow-up of IMMRP

Measure		BMI	NRS	HADA	HADD	PA1	PA2	AEA	AG	OEA	PEA	SEA	TL	TA
BMI	Pearson Correlation	1	0.43	0.07	-0.10	-0.25	0.02	0.42	0.21	-0.17	0.08	-0.12	-0.11	-0.09
	Sig. (2-tailed)		0.07	0.80	0.68	0.39	0.95	0.09	0.48	0.51	0.75	0.63	0.68	0.74
NRS	Pearson Correlation		1	0.19	0.16	-0.13	-0.44	0.18	0.21	-0.32	-0.38	-0.11	0.05	0.14
	Sig. (2-tailed)			0.45	0.52	0.67	0.11	0.47	0.47	0.20	0.12	0.66	0.85	0.60
HADA	Pearson Correlation			1	0.76**	-0.11	-0.02	0.08	-0.28	-0.03	0.11	-0.07	-0.18	-0.24
	Sig. (2-tailed)				0.00	0.72	0.94	0.76	0.33	0.90	0.66	0.79	0.49	0.35
HADD	Pearson Correlation				1	-0.34	0.07	-0.04	-0.27	-0.03	-0.01	-0.26	-0.21	-0.11
	Sig. (2-tailed)					0.23	0.81	0.87	0.36	0.90	0.97	0.29	0.41	0.69
PA1	Pearson Correlation					1	0.13	-0.45	-0.05	-0.11	-0.16	0.43	0.15	0.17
	Sig. (2-tailed)						0.65	0.11	0.88	0.72	0.60	0.13	0.61	0.59
PA2	Pearson Correlation						1	-0.56*	0.06	-0.25	-0.26	-0.16	-0.35	0.12
	Sig. (2-tailed)							0.04	0.86	0.40	0.38	0.58	0.22	0.70
AEA	Pearson Correlation							1	0.21	0.52*	.50*	-0.15	0.16	-0.17
	Sig. (2-tailed)								0.47	0.03	0.03	0.54	0.54	0.52
2-AG	Pearson Correlation								1	-0.20	0.07	0.08	-0.11	0.70**
	Sig. (2-tailed)									0.50	0.82	0.78	0.70	0.01
OEA	Pearson Correlation									1	.48*	-0.17	0.25	-0.32
	Sig. (2-tailed)										0.04	0.49	0.32	0.21
PEA	Pearson Correlation										1	0.38	-0.14	-0.26
	Sig. (2-tailed)											0.12	0.58	0.31
SEA	Pearson Correlation											1	-0.25	-0.16
	Sig. (2-tailed)												0.32	0.55
TL	Pearson Correlation												1	0.33
	Sig. (2-tailed)													0.20
TA	Pearson Correlation													1

_Supplementary Table S3 - Bivariate correlations: Δ-Correlations (_Pre-Post _IMMRP)_

**Measure**		ΔNRS	ΔHADA	ΔHADD	ΔPA1	ΔPA2	ΔAEA	Δ2AG	ΔOEA	ΔPEA	ΔSEA	ΔTL	ΔTA
ΔNRS	Pearson Correlation	1	0.03	0.43	-0.20	-0.14	0.20	0.36	-0.25	-0.32	-0.47*	0.17	0.28
	Sig. (2-tailed)		0.90	0.07	0.42	0.58	0.43	0.15	0.32	0.20	0.05	0.49	0.28
ΔHADA	Pearson Correlation		1	0.52*	0.17	-0.03	0.13	0.03	-0.01	-0.12	-0.03	0.02	0.15
	Sig. (2-tailed)			0.03	0.50	0.90	0.60	0.90	0.97	0.62	0.91	0.94	0.56
ΔHADD	Pearson Correlation			1	0.02	-0.02	0.01	-0.17	0.11	-0.09	-0.01	0.06	0.21
	Sig. (2-tailed)				0.92	0.93	0.96	0.49	0.67	0.73	0.95	0.80	0.41
ΔPA1	Pearson Correlation				1	0.70**	-0.50*	0.21	-0.14	-0.08	0.06	-0.02	0.41
	Sig. (2-tailed)					0.00	0.04	0.41	0.58	0.74	0.81	0.94	0.10
ΔPA2	Pearson Correlation					1	-0.58*	-0.09	-0.10	-0.03	0.17	-0.21	0.01
	Sig. (2-tailed)						0.01	0.73	0.69	0.90	0.50	0.40	0.98
ΔAEA	Pearson Correlation						1	0.03	0.45	0.54*	-0.06	-0.01	0.20
	Sig. (2-tailed)							0.916	0.062	0.022	0.82	0.982	0.429
Δ2AG	Pearson Correlation							1	-0.50*	-0.23	-0.25	0.18	0.38
	Sig. (2-tailed)								0.04	0.36	0.31	0.47	0.13
ΔOEA	Pearson Correlation								1	0.69**	0.18	0.14	-0.16
	Sig. (2-tailed)									0.00	0.47	0.57	0.54
ΔPEA	Pearson Correlation									1	0.45	-0.16	-0.06
	Sig. (2-tailed)										0.06	0.52	0.83
ΔSEA	Pearson Correlation										1	-0.52*	-0.31
	Sig. (2-tailed)											0.03	0.22
ΔTL	Pearson Correlation											1	0.27
	Sig. (2-tailed)												0.29
ΔTA	Pearson Correlation												1

Supplementary Tables S1-S3

Biviriate correlations of age (not in A2 and A3). BMI (not in A3). self-assessed pain intensity (NRS) and physical activity 1 and 2 (PA1 and PA2). The Hospital Anxiety (HADA) and Depression (HADD) Scale. Arachidonoylethanolamide (AEA). 2-arachidonoylglycerol (2AG). palmitoylethanolamide (PEA). oleoylethanolamide (OEA). stearoylethanolamide (SEA) in nM. telomer length (TL) in t/S ratio. and telomerase activity (TA) in TPG units/ µL. * implies $P \leq 0.05$ and ** $P \leq 0.01$

S1: Correlations Pre the interdisciplinary multimodal rehabilitation program (IMMRP)

S2 Correlations 6-month post IMMRP

S3: Δ-correlations = values pre IMMRP – values post IMMRP
